# Early and widespread injury of astrocytes in the absence of demyelination in acute haemorrhagic leukoencephalitis

**DOI:** 10.1186/2051-5960-2-52

**Published:** 2014-05-08

**Authors:** Christopher A Robinson, Reginald C Adiele, Mylyne Tham, Claudia F Lucchinetti, Bogdan FGh Popescu

**Affiliations:** Department of Pathology and Laboratory Medicine, Saskatoon Health Region/College of Medicine, University of Saskatchewan, 103 Hospital Drive, Saskatoon, SK S7N 0W8 Canada; Department of Anatomy and Cell Biology, College of Medicine, University of Saskatchewan, 701 Queen Street, Saskatoon, SK S7N 5E5 Canada; Cameco MS Neuroscience Research Center, University of Saskatchewan, Saskatoon City Hospital, 701 Queen Street, Saskatoon, SK S7K 0M7 Canada; Department of Neurology, Mayo Clinic, College of Medicine, 200 First Street SW, Rochester, MN 55905 USA

**Keywords:** Hurst’s disease, Aquaporin, Glial fibrillary acidic protein, Edema, Haemorrhage

## Abstract

Acute hemorrhagic leukoencephalitis (AHL) is a fulminant demyelinating disease of unknown etiology. Most cases are fatal within one week from onset. AHL pathology varies with the acuteness of disease. Hemorrhages, vessel fibrinoid necrosis, perivascular fibrin exudation, edema and neutrophilic inflammation are early features, while perivascular demyelination, microglial foci and myelin-laden macrophages appear later. Reactive astrocytosis is not present in early hemorrhagic non-demyelinated lesions, but is seen in older lesions. This case report presents the pathology of an AHL case with fulminant course and fatal outcome within 48 hours from presentation. Severe hemorrhages, edema and neutrophilic inflammation in the absence of circumscribed perivascular demyelination affected the temporal neocortex and white matter, hippocampus, cerebellar cortex and white matter, optic chiasm, mammillary bodies, brainstem, cranial nerve roots and leptomeninges. Perivascular end-feet and parenchymal processes of astrocytes exhibited impressive swelling in haemorrhagic but non-demyelinated white matter regions. Astrocytes were dystrophic and displayed degenerating processes. Astrocytic swellings and remnants were immunoreactive for aquaporin-4, aquaporin-1 and glial fibrillary acidic protein. These morphological changes of astrocytes consistent with injury were also observed in haemorrhagic and normal appearing cortex. Our findings reinforce that perivascular demyelination is not present early in AHL. This is the first study that highlights the early and widespread astrocytic injury in the absence of demyelination in AHL, suggesting that, similarly to neuromyelitis optica and central pontine myelinolysis, demyelination in AHL is secondary to astrocyte injury.

## Introduction

Acute hemorrhagic leukoencephalitis (AHL), or Hurst’s disease, is a fulminant demyelinating disease of unknown etiology with fatal outcome within one week from onset due to severe cerebral edema and herniation [1, 2]. Histopathologically, AHL is characterized by the presence of perivascular haemorrhagic demyelinating lesions with prominent edema, axonal injury and predominantly neutrophilic parenchymal and meningeal inflammatory infiltrates [1, 2]. Based on the pathological similarities between acute disseminated encephalomyelitis (ADEM) and AHL, AHL is now considered a hyperacute variant of ADEM [3]. In many cases, both AHL and ADEM occur within 2 to 4 weeks of a premonitory infection, most commonly a viral upper respiratory infection, although the prerequisite of an antecedent infection is neither specific nor sensitive for their diagnosis [1, 4, 5].

The pathological features of AHL differ between acute and late disease stages [6, 7]. Severe hemorrhages, fibrin impregnation of blood vessel walls and perivascular fibrin exudation, edema and predominantly neutrophilic inflammation are characteristic for the early AHL stages seen in patients with fulminant disease and fatal course within 2 days. Perivascular demyelination, perivascular microglial foci and myelin-laden macrophages appear later in the disease evolution as seen in patients with a longer disease course. Similarly, hypertrophic reactive astrocytes are not seen in early hemorrhagic non-demyelinated lesions, but may become apparent in older AHL lesions [7, 8]. Herein, we describe the pathology of a case of AHL with a fulminant and fatal course, and provide histopathological evidence that damage to astrocytes is an early event that precedes demyelination in AHL.

### Consent

This study was approved by the University of Saskatchewan Biomedical Research Ethics Board (Bio-REB # 11–217). The Bio-REB issued a waiver of the requirement of consent for the examination of retrospective archival pathological material when patient or next of kin contact was not possible due to unavailable medical records and contact information (this is a 12 year old case). All samples were deidentified. Bio-REB considered the requirements of section 29 under the Health Information Protection Act (HIPA) and was satisfied that this study meets the privacy considerations outlined therein.

## Case report

A 39 year old male patient presented to the ER department for new-onset seizures and severe headache preceded by three days of flu-like symptoms. A CT head was performed but revealed no abnormalities. The patient was prescribed sedatives and discharged home. The next day the patient became lethargic and slow to answer questions. Subsequently his level of consciousness deteriorated rapidly and he was found unconscious when Emergency Medical Services arrived. He was treated on site for narrow complex tachycardia (160/min) without any benefit and then transported to the ER. Neurological examination revealed deep coma (Glasgow Coma Scale 3/15) with pinpoint pupils. His general examination was remarkable for tachycardia (160/min), hypertension (214/116) and few respiratory crackles. He was intubated for airway protection. A CT scan revealed mild dilation of the ventricular temporal horns and poor grey – white matter differentiation. A chest X-ray was suggestive of pulmonary edema. An ECG revealed atrial flutter with 2:1 block that responded to amiodarone infusion. CBC showed 17.5X10^9^ white blood cells/L (differential not available), normal haemoglobin and platelet counts. Drug screen and blood cultures were negative. CSF examination revealed bloody fluid, with increased protein (4.66 g/l) and 365 cells/μl with 75% lymphocytes and 25% polymorphonuclear leukocytes. CSF Gram stain and cultures were negative.

The patient was admitted to ICU where he reverted to narrow complex tachycardia and became hypotensive. He received DC shocks multiple times and the amiodarone bolus was repeated. The patient reverted to sinus rhythm, but remained hypotensive, and IV fluids and vasopressors were administered. He continued to deteriorate, went into a ventricular rhythm and was noted to have fixed dilated pupils. Despite cardiovascular support, he became asystolic and cardiopulmonary resuscitation proved unsuccessful. The patient was pronounced dead 3 hours after admission.

### Pathology

At autopsy, the brain was markedly swollen and weighed 1650 g. Gross examination revealed moderate cortical gyral flattening, and bilateral uncal and cerebellar tonsil herniation. Diffuse, spotty haemorrhages were noted in the temporal lobes, cerebellar hemispheres and brainstem. The large intracranial arteries at the base of the brain were normal in calibre and distribution, and showed no pathological changes. There was no evidence of aneurysm and no free blood in the cranial cavity. The spinal cord was unremarkable both grossly and microscopically.

Brain tissue was fixed in 10–15% formalin and embedded in paraffin. Sections, 5 μm thick, were stained with haematoxylin and eosin (HE) for morphological evaluation and Luxol-fast blue- haematoxylin and eosin (LFB/HE) to demonstrate myelin. Immunohistochemistry was performed using an avidin–biotin technique without modification [9]. Antigen retrieval was performed as previously described. Tissues were exposed (16 hrs, at 4°C), to primary antibodies specific for: aquaporin-1 (AQP1; rabbit polyclonal 1:500; Santa Cruz, USA), aquaporin-4 (AQP4; affinity-purified rabbit polyclonal 1:250; Sigma-Aldrich, USA), glial fibrillary acidic protein (GFAP, mouse monoclonal 1:4000; Dako, Denmark), myelin proteolipid protein (PLP, rabbit polyclonal 1:500; Serotec, Oxford, USA), myelin oligodendrocyte glycoprotein (MOG, rabbit monoclonal 1:1000; Abcam, USA), myelin-associated glycoprotein (MAG, rabbit monoclonal 1:500; Sigma, USA), T lymphocytes (CD3, rat monoclonal 1:400; Serotec, USA), cytotoxic T lymphocytes (CD8, mouse monoclonal 1:50; Dako, Denmark), macrophages/microglial cells (CD68, mouse monoclonal 1:1000; Dako, Denmark), human immunoglobulin G (IgG, rabbit monoclonal 1:500; Epitomics, USA), herpes simplex virus 1 and 2 (HSV 1&2, rabbit polyclonal 10 mg/ml; Biocare Medical, USA) and cytomegalovirus (CMV, mouse monoclonal, 10 mg/ml; Biocare Medical, USA). Primary antibodies were omitted in control staining. In situ hybridization was performed using fluorescein-labeled oligonucleotide probes specific for Epstein-Barr Virus-Encoded RNA (EBER; Ventana Medical Systems, USA).

**Figure 1:**
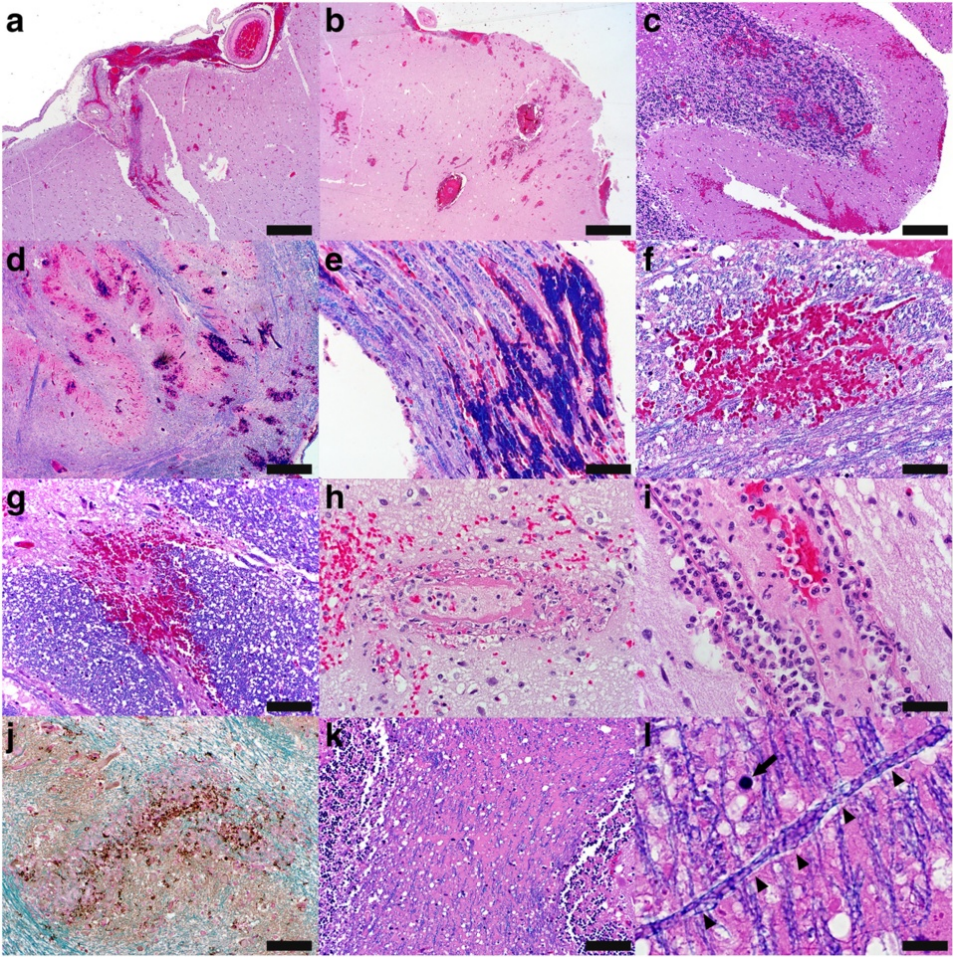
**Neuropathology of AHL.** Haemorrhages are present in the **(a)** leptomeninges (HE, scale bar = 500 μm), **(b)** temporal lobe (HE, scale bar = 1600 μm), **(c)** cerebellum (HE, scale bar = 200 μm), **(d)** medullary olive (LFB/HE, scale bar = 500 μm) and **(e)** vagus nerve root (LFB/HE, scale bar = 100 μm). **(f)** Ball (LFB/HE, scale bar = 100 μm) and **(g)** ring haemorrhages (LFB/HE, scale bar = 100 μm) affect the pontine white and gray matter, respectively. **(h)** An involved blood vessel shows evidence of fibrinoid necrosis and fibrin exudation (HE, scale bar = 50 μm). **(i)** Perivascular inflammatory infiltrates consist mainly of neutrophils (HE, scale bar = 33 μm). **(j)** Parenchymal microglial foci are confined to haemorrhagic areas [CD68/LFB/Nuclear Fast Red (NFR), scale bar = 100 μm]. **(k)** Myelin pallor is present in the cerebellar white matter and seems to be due to spreading of the myelin sheaths because of edema and diffusion of an eosinophilic fibrin-like substance conferring the white matter a sieve-like appearance (LFB/HE, scale bar = 100 μm). **(l)** High magnification visualization of the myelinated fibers reveals vacuolation and decompaction of myelin (arrow heads); rare apoptotic oligodendrocytes are present (arrow) (LFB/HE, scale bar = 33 μm).

Microscopically, perivascular haemorrhages, involving small veins and venules preferentially, were present and abundant in the leptomeninges (Figure 1a), neocortex and subcortical white matter of the temporal lobes (Figure 1b), hippocampus, cerebellar cortex and white matter (Figure 1c), optic chiasm, mammillary bodies, throughout the brainstem (Figure 1d, f-g) and in the cranial nerve roots (Figure 1e). Within the medulla, hemorrhages involved the floor of the fourth ventricle bilaterally, while the inferior olivary nucleus, olivocerebellar fibers, amiculum olivae and the pyramids were affected unilaterally (Figure 1d). Ball and ring haemorrhages affected grey and white matter indiscriminately throughout the nervous system both supra- and infratentorially (Figure 1f,g). In many instances, involved vessels showed evidence of fibrinoid necrosis and fibrin exudation (Figure 1h). Occasionally the vessel lumen was occluded by fibrin or platelet thrombi. Moderate and marked inflammatory infiltrates were present perivascularly, within the vessel walls and parenchymally. They consisted mainly of neutrophils (Figure 1i). Mild and moderate perivascular and parenchymal invasion with monocyte and T-lymphocyte, predominantly cytotoxic T-cells, was also present. Perivascular microglial foci were rare. Parenchymal microglial nodules were present but restricted to hemorrhagic areas (Figure 1j). There was no evidence of perivascular immunoglobulin deposition. Neither reactive astrocytosis nor neuronal eosinophilia were present.

Apart from the presence of hemorrhages, edema with tissue vacoulation was the most prominent neuropathological feature observed both at gross examination and microscopically. Myelin was well preserved and perivascular demyelination not present. Myelin pallor was present in the inferior olive and cerebellar white matter and appeared to be due to spreading of the myelin sheaths because of edema and diffusion of an eosinophilic fibrin-like substance similar to the perivascular fibrin exudates conferring the white matter a sieve-like appearance (Figure 1k,l). Higher magnification of the myelinated fibers in these areas of myelin rarefaction revealed vacuolation and decompaction of myelin (Figure 1l, arrowheads). Rare apoptotic oligodendrocytes were present (Figure 1l, arrow).

**Figure 2:**
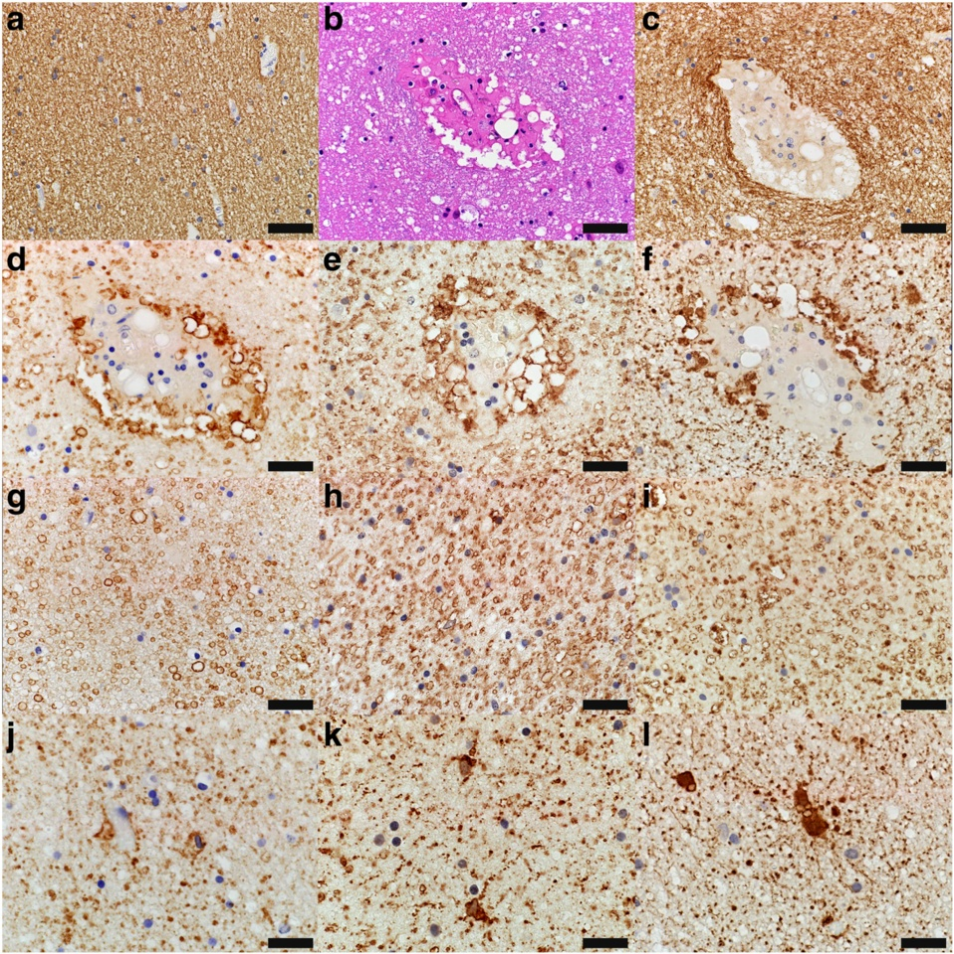
**Astrocytes in the affected white matter show morphological changes consistent with injury in the absence of demyelination. (a)** Myelin is preserved in white matter regions affected by haemorrhages (PLP, scale bar = 50 μm). **(b)** White matter blood vessel shows perivascular fibrin exudation (also note the absence of axonal swellings consistent with absence of axonal injury) (HE, scale bar = 50 μm), but **(c)** perivascular myelin is preserved (PLP, scale bar = 50 μm). **(d-f)** Perivascular astrocyte end-feet exhibit impressive swelling: astrocytic swellings are immunoreactive for **(d)** AQP4 (AQP4, scale bar = 50 μm), **(e)** AQP1 (AQP1, scale bar = 50 μm) and **(f)** GFAP (scale bar = 50 μm). **(g-i)** Parenchymal astrocyte processes exhibit impressive swelling: astrocytic swellings are immunoreactive for **(g)** AQP4 (AQP4, scale bar = 50 μm), **(h)** AQP1 (AQP1, scale bar = 50 μm) and **(i)** GFAP (scale bar = 50 μm). **(j-l)** Astrocytes are dystrophic and have “beaded” processes consistent with degeneration: the dot-like astrocytic remnants are immunoreactive for **(j)** AQP4 (AQP4, scale bar = 33 μm), **(k)** AQP1 (AQP1, scale bar = 33 μm) and **(l)** GFAP (scale bar = 33 μm).

Astrocytes in these non-demyelinated white matter regions (Figure 2a,c) affected by hemorrhages and perivascular fibrin exudates (Figure 2b) displayed morphological changes consistent with injury (Figure 2d-l). Perivascular astrocyte end-feet and parenchymal astrocyte processes exhibited impressive swelling (Figure 2d-i). Astrocytes were dystrophic and displayed “beaded” processes consistent with degeneration (Figure 2j-l). Both the astrocytic swellings and the dot-like astrocytic remnants were immunoreactive for AQP4, AQP1 and GFAP (Figure 2d-l).

**Figure 3:**
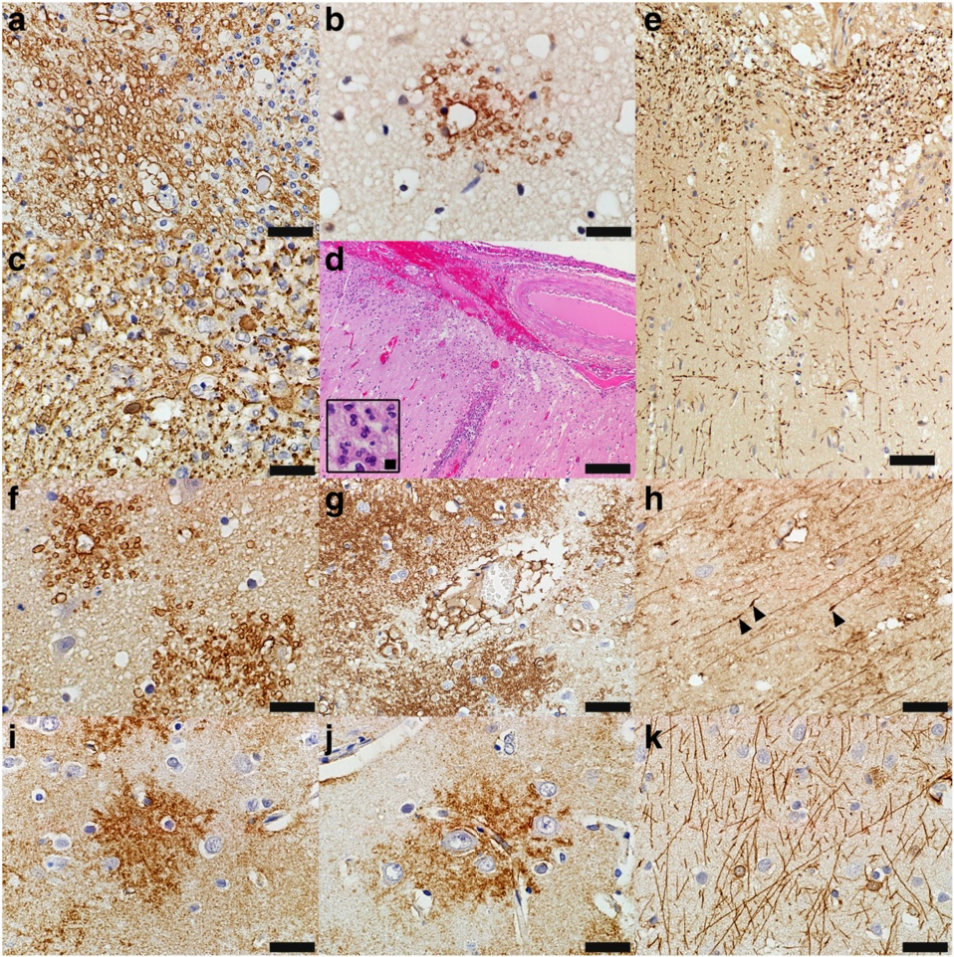
**Astrocytes in the affected and non-affected cortices are injured. (a-b)** Astrocyte swellings are immunoreactive for **(a)** AQP4 (AQP4, scale bar = 50 μm)) and **(b)** AQP1 (AQP1, scale bar = 33 μm). **(c)** Degenerated astrocyte processes are immunoreactive for GFAP (GFAP, scale bar = 33 μm). **(d)** Meningeal haemorrhages and inflammation extend into the perivascular spaces and upper cortical layers (HE, scale bar = 200 μm); inset shows neutrophils infiltrating into the molecular layer of the cortex (HE, scale bar = 8.75 μm). **(e)** Cortical myelin is preserved in regions affected by haemorrhage (PLP, scale bar = 100 μm). **(f-g)** Astrocytes in the frontal cortex which does not show haemorrhages or vascular changes have **(f)** swollen processes (AQP4, scale bar = 33 μm) and **(g)** swollen perivascular end-feet (AQP4, scale bar = 50 μm). **(h)** Astrocyte swellings in the frontal cortex also display GFAP immunoreactive swellings (GFAP, scale bar = 33 μm). **(i-k)** Normal morphology of astrocytes in the occipital cortex of this case: **(i)** parenchymal astrocytes (AQP4, scale bar = 33 μm); **(j)** perivascular astrocytes (AQP4, scale bar = 50 μm); and **(k)** astrocytic fibers (GFAP, scale bar = 33 μm).

Despite the presence of meningeal hemorrhage and inflammation often extending in the Virchow–Robin spaces (Figure 3d), tissue vacuolation, parenchymal hemorrhages and neutrophilic infiltrates, the myelin in the temporal cortex was preserved (Figure 3e). However, protoplasmic astrocytes exhibited both swelling and degeneration of their processes and perivascular end-feet (Figure 3a-c), and the severity of these changes decreased from the pia toward the white matter. Vacuolation of protoplasmic astrocytic processes (Figure 3f) and perivascular end-feet (Figure 3g), as well as GFAP-positive astrocytic swellings (Figure 3h) were also present in the frontal cortex in the presence of meningeal inflammation, but in the absence of either perivascular or parenchymal brain tissue inflammatory infiltrates, or vasculopathy. Astrocytes in the parietal and occipital cortex displayed normal morphology (Figure 3i-k).

Tissue stains for microorganisms, including HSV, EBV, CMV and fungi were negative. No viruses were isolated in the CSF collected at necropsy and the cryptococcal antigen test was negative.

Microscopic examination of the myocardium of the left ventricle and posterior wall of the right ventricle revealed the presence of polymorphonuclears, predominantly perivascularly. There was no evidence of necrosis, fibrosis or damage to the vessel wall, and no evidence of damage to the myocytes. Sections of the lungs revealed pulmonary edema, and there was acute congestion of the liver. Examination of other organs and tissues reveled no pathological changes.

## Discussion

While haemorrhagic perivascular demyelination is considered the histopathologic hallmark of AHL, previous studies have shown that the pathology of AHL varies with the acuteness of the disease [6, 7]. The predominance and severity of haemorrhages and edema, and the preponderance of neutrophils in inflammatory infiltrates we have found in this case coupled with the absence of frank perivascular demyelination, the absence of macrophages and the rarity of perivascular microglial infiltrates are acute histopathological features consistent with the short interval between onset and death in AHL [6], and similar to what is observed in AHL experimental models [10]. In cases with a longer course, increasing proportions of macrophages and microglia are found in the inflammatory infiltrates, and perivascular demyelination may become obvious [6]. Our findings corroborated with previously published studies reinforce the concept that disease stage influences AHL pathology: circumscribed perivascular demyelination is not present in fulminant cases with fatal outcome within 1 to 2 days from onset.

This is the first study that highlights the early and widespread injury of astrocytes in AHL. We show that both protoplasmic and fibrous astrocytes exhibit swelling of their end-feet and degeneration of their processes and cell bodies in the absence of demyelination and considerable oligodendrocyte injury, suggesting that astrocytes may be an early initial target in AHL and that demyelination is secondary. These findings may also explain the absence of reactive astrocytosis seen in this case, and other reported acute AHL cases [8]. While we show that astrocytes are the first nervous cells affected in AHL, it is possible that primary vasculopathy with secondary tissue destruction-related neutrophil infiltration and vasogenic edema causes astrocytic injury.

However, we also found astrocytes with swollen processes and perivascular end-feet in cortical regions without hemorrhages, brain tissue inflammation or vascular changes. This reinforces the early astrocytic disturbance in AHL that may be independent of the presence of vascuolpathy, and raises interesting questions regarding the chronology of lesion development. Morphological changes of astrocytes similar to those described here have been reported in cytotoxic edema associated with hyponatremia, ischemia, brain trauma and hepatic encephalopathy [11–14]. Furthermore, imaging studies have shown that decreased apparent diffusion coefficient consistent with cytotoxic edema is present in AHL before the blood–brain barrier (BBB) is altered [15, 16]. All these findings show an important early role for cytotoxic edema in AHL and suggest that hypoxic, osmotic or toxic stress may drive the pathogenesis of this idiopathic disorder and/or render these central nervous system (CNS) regions more susceptible to BBB damage, free entry of blood toxins or autoantibodies, and development of lesions [17].

Meningeal inflammation is a constant pathological finding in ADEM and AHL [1, 6–8], and is the earliest histopathological feature observed in experimental allergic encephalomyelitis (EAE) and hyperacute EAE [10, 18], preceding the appearance of parenchymal inflammation and demyelination. Furthermore, we show that the upper cortical layers located in close proximity to the haemorrhagic and inflamed leptomeninges exhibit severe astrocytic fragmentation, while the further located deep cortical layers only show astrocyte swelling. It is therefore plausible that the cytotoxic swelling of astrocytes with or without breakdown of BBB may be caused by toxic products released by neutrophils and other inflammatory cells that have infiltrated the subarachnoid space [19], and suggest a potential beneficial role for neutrophil-depletion therapies in AHL [19, 20].

Both neuromyelitis optica (NMO) and central pontine myelinolysis (CPM) are primary astrocytopathies with secondary demyelination [21–23]. NMO is an autoimmune inflammatory astrocytopathy caused by anti-AQP4 complement-activating IgG autoantibodies [24, 25], while astrocytic injury in CPM is a consequence of the osmotic stress [22]. Early lesions in both NMO and a subgroup of CPM patients are characterized by astrocytic damage, and loss of AQP4 in NMO and of both AQP4 and AQP1 in CPM [22, 23]. The BBB is altered in both NMO (required for the entry of the pathogenic autoantibodies into the CNS) and CPM (caused by endothelial shrinkage due to the rapid correction of chronic hypotonicity with a hyperosmolar solution). Loss of AQP4 in NMO is caused by its internalization by astrocytes or by complement-mediated destruction of astrocytes following binding of NMO-IgG to AQP4 [26], while loss of AQP4 and AQP1 in CPM may represent a protective mechanism whereby astrocytes restrict water loss to prevent apoptosis [22]. The disruption of the BBB coupled with the inability of astrocytes to either buffer or eliminate the excess water in NMO and CPM causes vasogenic edema and increased osmolarity of the CNS extracellular space and may trigger and/or exacerbate the intramyelinic edema, oligodendrocyte apoptosis and secondary demyelination. Early AHL lesions exhibit preservation of AQP4 and AQP1. Aquaporins represent the rate-limiting step for water flow from the vascular compartment into the CNS [27]. Therefore, the preservation of AQP1 and AQP4 allows astrocytes to initially buffer the water that flows from the blood into the CNS through an intact BBB likely due to hypoosmolality or hypoxic energy failure that causes cytotoxic edema [27]. However, in the presence of a persisting insult, the compensatory mechanisms ultimately fail and the swollen astrocytes die [28].

In conclusion, we present a case of AHL with rapid progression and fatal outcome within less than 48 hours from onset, whose neuropathologic picture is dominated by the widespread swelling and degeneration of astrocytes in the absence of demyelination, suggesting that, similarly to NMO and CPM, demyelination in AHL is secondary to astrocyte injury.
